# Molecular variation of tomato yellow leaf curl virus in the insect vector *Bemisia tabaci*

**DOI:** 10.1038/s41598-017-16330-4

**Published:** 2017-11-27

**Authors:** Xiuling Yang, Bi Wang, Junbo Luan, Yan Xie, Shusheng Liu, Xueping Zhou

**Affiliations:** 1 State Key Laboratory for Biology of Plant Diseases and Insect Pests, Institute of Plant Protection, Chinese Academy of Agricultural Sciences, Beijing, 100193 China; 2 State Key Laboratory of Rice Biology, Institute of Biotechnology, Zhejiang University, Hangzhou, 310058 China; 3 Institute of Insect Sciences, Zhejiang University, Hangzhou, 310058 China

## Abstract

Insect vectors play significant roles in geminivirus spread and evolution in nature. To date little is known about the population dynamics of begomoviruses in their insect vector *Bemisia tabaci*. In this study we analyzed the genetic variation of tomato yellow leaf curl virus (TYLCV) in its host plant, *Solanum lycopersicum*, in its transmission vector *B. tabaci* raised on TYLCV-infected *S. lycopersicum* plants, and in *B. tabaci* after being transferred from *S. lycopersicum* to *Gossypium hirsutum*. We found that the levels of variability of TYLCV remained stable in *S. lycopersicum* plants, but increased significantly in both invasive and indigenous species of *B. tabaci*. We also presented evidence that the elevated mutation frequencies in TYLCV populations from vector whiteflies were caused mainly by mutations that occurred at several distinct sites within the TYLCV genome. Simultaneous introduction of mutations in the hot spots did not affect the ability of TYLCV to be transmitted by *B. tabaci*, but reduced its pathogenicity in both *S. lycopersicum* and *Nicotiana benthamiana*. Our findings provide new information on population variability of TYLCV in its insect vector, extending the knowledge of the influence of insect vector on plant virus population dynamics.

## Introduction

Mutations that occur during virus replication are one of the main sources of genetic variation in viruses. Together with natural selection, genetic drift, gene flow and recombination, the genetic makeup of virus populations in their hosts can change at a great speed^1^. Consequently, analysis of genetic structure and evolution of virus populations is crucial to our understanding of the molecular mechanisms underlying virus evolution, emergence of new viruses and their epidemiology in nature. Information obtained from studies on virus evolution will facilitate us to develop more efficient and more durable strategies for virus disease management.

RNA viruses have been shown to have high potential for genetic variation due mainly to the error-prone replication by viral RNA-dependent RNA polymerases. A single replicating population of an RNA virus can have genetically diverse populations known as quasispecies, and the mutation rates for RNA viruses were estimated at 10^−3^ to 10^−5^ substitutions per site per year (subs/site/year)^2,3^. The mechanism underlying viral genetic variability is complex and is determined by multiple virus- and host-dependent processes^4,5^. Different steps of the life cycle of RNA viruses, such as vector-mediated transmissions and systemic colonization of new leaves, may impose a bottleneck^6–8^. These bottlenecks could have profound effects on the maintenance of genetic variation of virus populations, thereby ultimately determing the extent to which different random forces drive virus evolution. While genetic drift is the major force acting during evolution, fitness is reported to substantially decrease as a consequence of the onset of Muller’s ratchet^7^.

Geminiviruses are important plant DNA viruses that have been reported to infect a wide range of crops, including both monocotyledonous and dicotyledonous species. Geminiviruses are currently classified into nine genera (*Becurtovirus*, *Begomovirus*, *Capulavirus*, *Curtovirus*, *Eragrovirus*, *Grablovirus*, *Mastrevirus*, *Topocuvirus*, and *Turncurtovirus*) based on their genome structures, insect vectors, and host range^9^. *Begomovirus* represents the largest genus within the family *Geminiviridae* and has over 320 species^9^. Begomoviruses are transmitted by *Bemisia tabaci* (whitefly) in a persistent and circulative manner, and use DNA polymerases of their host plants to replicate their genomic DNAs inside the nucleus^10,11^. Plant DNA polymerase was previously reported to have multiple functions, including the error-correcting activity. This error-correcting activity can reduce the mutation rate during DNA virus replication by at least an order of magnitude^12^. However, presence of high intrapopulation diversity in begomoviruses and maize streak virus has been reported previously^13–16^. The genetic structure and variability of begomovirus populations from a naturally infected tomato plant and from experimentally infected tomato and *Nicotiana benthamiana* plants generated comparable diversity levels to those reported for plant RNA viruses^13^. However, direct estimations of population dynamics of begomoviruses in its whitefly vector *B. tabaci* is lacking.

Tomato yellow leaf curl virus (TYLCV) in the genus *Begomovirus* of the family *Geminiviridae* is one of the devastating viruses causing tomato yellow leaf curl disease (TYLCD). It was originally described in the Middle East in the 1960’s, and has now spread to tropical, subtropical and temperate regions of the world. In China, it was first identified in Shanghai in 2006^17^. Since then, TYLCD outbreaks have been reported in 15 provinces and have caused severe damage to tomato production in China^18^. Like other begomoviruses, TYLCV is transmitted by the whitefly *Bemisia tabaci* in a circulative manner. In a previous study, both invasive *B. tabaci* Mediterranean (MED) and the indigenous *B. tabaci* Asia II 1 have been shown to be able to retain TYLCV DNA for their entire adult life. The invasive *B. tabaci* MED, however, transmits TYLCV more efficiently than the indigenous *B. tabaci* Asia II 1^19^. In this study, we compared full-length genomic sequences of TYLCV obtained from TYLCV-infected *Solanum lycopersicum* (tomato) plants, from the invasive *B. tabaci* MED as well as the indigenous *B. tabaci* Asia II 1. By estimating the rates of nucleotide substitution and the distribution pattern of mutations, we found that the genetic variability of TYLCV populations changed in both species of whiteflies. In addition, introduction of mutations occurring in the TYLCV progeny obtained from whiteflies reduced the pathogenicity of TYLCV in both tomato and *N. benthamiana*.

## Results

### Genetic variability of TYLCV in experimentally-infected tomato plants

**Figure 1:**
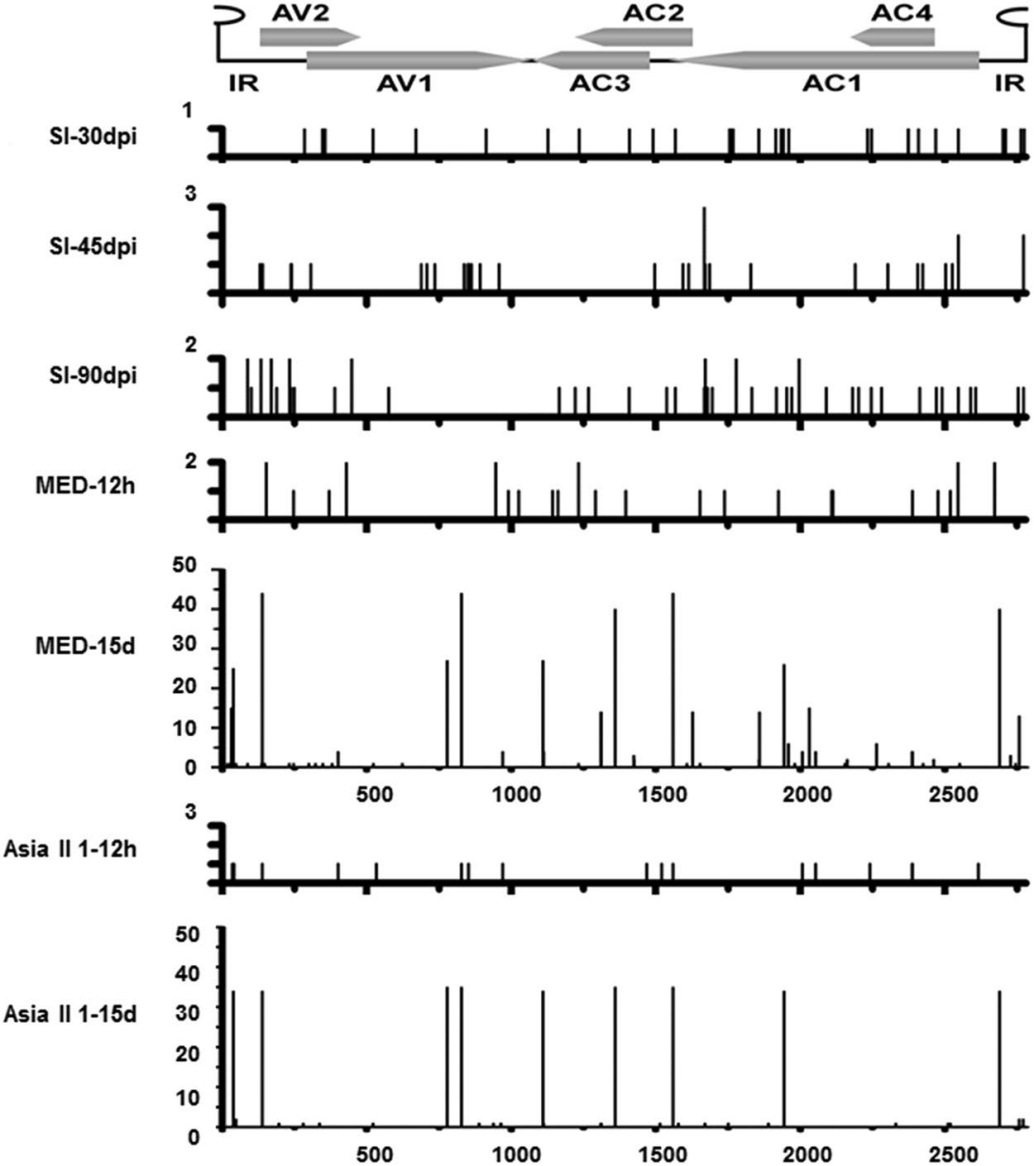
Distribution of mutations in TYLCV populations from the TYLCV-infected tomato and viruliferous *Bemisia tabaci*. The vertical lines indicate the number of mutations occurred at the indicated positions. The genomic organization of TYLCV is shown in the linear form. Sl-30 dpi, Sl-45 dpi and Sl-90 dpi represent sequences obtained from the TYLCV-infected tomato plants at 30 days, 45 days and 90 days post inoculation, respectively. MED-12 h and Asia II 1–12 h represent sequences obtained from the *B. tabaci* MED and Asia II 1 whiteflies collected after 12 h acquisition access period, respectively. MED-15 d and Asia II 1–15 d represent sequences obtained from the *B. tabaci* MED and Asia II 1 whiteflies collected at 15 d after being transferred from the infected tomato to cotton plants, respectively.

High genetic variability has been documented in natural populations of TYLCV^20^. To determine the genetic variability of TYLCV in an experimentally controlled environment, we first analyzed the variability of TYLCV progeny populations from systemically infected tomato leaf tissues at 30, 45 and 90 days post inoculation. Three tomato plants were sampled at each time point, and full-length viral sequences from each sample were cloned separately. Each clone represented a unique viral DNA, and approximately 60 clones from three different biological replicates were randomly selected to represent the TYLCV populations from the infected tomato plants. Comparison of the full-length sequences of the viral clones to the parental sequence that initiated the infection showed that the levels of genetic variation estimated by mutation frequency and the percentage of mutated viral clones, as well as the consensus sequence in TYLCV progeny populations, were stable in infected tomato plants over the time course of the study. Mutations observed in the populations from the TYLCV-infected tomato plants at various dpi were distributed randomly across the viral genome, with the levels of genetic variation ranging from 2.02 × 10^−4^ to 3.66 × 10^−4^ (Fig. 1, Table 1). This variation was, however, not statistically significant between the sampled plants at the three collection time (Table 1), but was significantly lower than the mutation rate introduced by the process of rolling-cirlce amplification and sequencing effort, which was determined to be 1.8 × 10^−5^ (Table 1).

**Table 1:** Variation in TYLCV populations obtained from the TYLCV-infected tomato and viruliferous *B. tabaci* MED and *B. tabaci* Asia II 1.

Host	Days p.i.	Replicate	% of mutant clone	Total mutations/bases sequenced	Mutation frequency^*b*^
tomato	30	1	29.4% (5/17)	5/47277	1.06 × 10^−4^
		2	40% (8/20)	9/55620	1.62 × 10^−4^
		3	52.9% (9/17)	16/47277	3.38 × 10^−4^
		Total	40.77%	30/150174	2.02 × 10 ^−4^ a
	45	1	58.8% (10/17)	11/47277	2.32 × 10^−4^
		2	50% (9/18)	14/50058	2.80 × 10^−4^
		3	61.1% (11/18)	12/50058	2.39 × 10^−4^
		Total	56.60%	37/147393	2.5 × 10 ^−4^ a
	90	1	70.6% (12/17)	21/47277	4.44 × 10^−4^
		2	64.7% (11/17)	13/47277	2.75 × 10^−4^
		3	66.7% (12/18)	19/50058	3.8 × 10^−4^
		Total	67.30%	53/144612	3.66 × 10 ^−4^ a
MED	0.5^a^	1	18.75% (3/16)	3/44496	6.74 × 10^−5^
		2	45% (9/20)	18/55620	3.23 × 10^−4^
		3	35% (7/20)	8/55620	1.43 × 10^−4^
		Total	32.92%	29/155736	1.78 × 10 ^−4^ a
	15	1	93.3% (14/15)	139/41715	3.33 × 10^−3^
		2	94.4% (17/18)	156/50058	3.12 × 10^−3^
		3	100% (15/15)	199/41715	4.77 × 10^−3^
		Total	95.9%	494/133488	3.74 × 10 ^−3^ b
Asia II 1	0.5^a^	1	33.3% (4/12)	15/33372	4.49 × 10^−4^
		2	7.69% (1/13)	1/36153	2.76 × 10^−5^
		3	4.54% (1/22)	1/61182	1.63 × 10^−5^
		Total	15.18%	17/130707	1.643 × 10 ^−4^ a
	15	1	100% (12/12)	117/33372	3.50 × 10^−3^
		2	100% (11/11)	104/30591	3.40 × 10^−3^
		3	100% (12/12)	114/33372	3.42 × 10^−3^
		Total	100%	335/97335	3.44 × 10 ^−3^ b
Rolling circle amplification control			5%(1/20)	1/55620	1.80 × 10 ^−5^c

^a^12 hours. ^b^Statistically significant differences in diversity levels are denoted by letters next to mutation frequencies. Mutation frequencies with the same letter are not statistically different. Least significant differences were determined using the ANOVA test (*p* ≤ 0.05 level).

### Genetic structure and variability of TYLCV populations in *B. tabaci* MED whiteflies

Previous study showed that viruliferous *B. tabaci* MED and Asia II 1 whitefly adults could retain TYLCV DNA for their whole life cycle after they acquired the virus from the infected plant^19^. Furthermore, TYLCV DNA was reported to be detected in whitefly adults after only 30-min acquisition access period (AAP) on TYLCV-infected plants, and the percentage of adults containing the viral DNA could be as high as 100% by 12 h AAP. To determine the variation of TYLCV populations in *B. tabaci*, and at the same time to minimize the effect of feeding procedure on the variation of TYLCV populations, we allowed *B. tabaci* MED whiteflies to feed on TYLCV-infected tomato plants showing typical TYLCV symptoms for 12 h. After feeding, approximately half of the whiteflies were collected and the other half of whiteflies were transferred to cotton, a previously reported non-host plant of TYLCV^21^, for another 15 days before DNA extraction.

Analysis of TYLCV populations obtained from three individual *B. tabaci* MED whiteflies at 12 h AAP showed that sequences identical to the parental TYLCV sequence were the dominant ones. The percentage of clones containing one or more mutations accounted for 32.92%, and the average mutation frequency in the *B. tabaci* MED whiteflies at 12 h AAP was 1.78 × 10^−4^ (Table 1). Interestingly, analysis of TYLCV populations obtained from *B. tabaci* MED whiteflies at 15 days after being transferred from the TYLCV-infected tomato to *Gossipium hirsutum* (cotton) plants showed that the majority of cloned sequences contained at least one nucleotide change compared with the parental sequence. The level of population diversity, estimated by the percentage of clones with at least one nucleotide change, and the mutation frequency reached 95.90% and 3.74 × 10^−3^, respectively (Table 1). Statistical analysis revealed that variation among TYLCV progeny populations from *B. tabaci* MED whiteflies collected at 15 days after being transferred to cotton plants were significantly higher than that observed in the progeny populations obtained from either *B. tabaci* MED whiteflies collected at 12 h AAP or from TYLCV-infected tomato plants (Table 1). These results show that after allowing *B. tabaci* MED whiteflies to feed on cotton plants for 15 days, the mutation frequencies of TYLCV populations increased significantly when compared with that of TYLCV maintained consistently in infected tomato plants.

### Distribution of mutations in *B. tabaci* MED whiteflies

To better understand the population dynamics of TYLCV in *B. tabaci* MED whiteflies, we compared the distribution of all the mutations identified in individual TYLCV progeny populations. Results of the comparisons showed that the mutations observed in the populations from TYLCV-infected tomato plants at 30, 45 and 90 dpi were distributed randomly across the viral genome (Fig. 1). Similar results were found in the populations from *B. tabaci* MED whiteflies collected at 12 h AAP from infected tomato plants. Several specific mutations were found to occur more frequently in the populations from *B. tabaci* MED whiteflies collected at 15 d after being transferred to cotton plants (Fig. 1). These include mutations at nucleotide positions 3, 31, 38, 138, 2689 and 2757 (within the intergenic region, IR), 778 and 828 (within the V1 ORF), 1110 (within the C2 ORF), 1310, 1311, and 1359 (within the C2-C3 overlapping region), 1559 and 1627 (within the C1-C2 overlapping region), and 1858, 1944 and 2031 (within the C1 ORF). These 17 mutations accounted for 71.37% of the total mutations identified in this study. The mutation frequencies in the IR, V1, V2, C1, C2, C3 and C4 ORFs were 1.09 × 10^−2^, 2.25 × 10^−3^, 5.93 × 10^−4^, 2.98 × 10^−3^, 8.37 × 10^−3^, 3.81 × 10^−3^ and 1.28 × 10^−3^, respectively. Except for the V2 ORF, the mutation frequencies observed in the IR and the other five other ORFs were much higher than those found in the TYLCV progeny populations obtained from infected tomato plants (Table 2). When the mutations occurring at the same nucleotide position were grouped and counted as a single mutation, the number of mutation sites is still higher than that in populations obtained from tomato and *B. tabaci* at 12 h AAP (data not shown).

**Table 2:** Comparison of variability within the intergenic region and six ORFs of TYLCV.

Host	Days p.i		IR	V1	V2	C1	C2	C3	C4
Tomato	30	Total mutations/bases sequenced	4/16902	5/41958	3/18954	16/57996	3/22032	3/21870	5/15876
		Mutation frequency	2.37 × 10^−4^	1.19 × 10^−4^	1.58 × 10^−4^	2.76 × 10^−4^	1.36 × 10^−4^	1.37 × 10^−4^	3.15 × 10^−4^
	45	Total mutations/bases sequenced	3/16589	11/41181	3/18603	19/56922	1/21624	0/21465	4/15582
		Mutation frequency	1.81 × 10^−4^	2.67 × 10^−4^	1.61 × 10^−4^	3.33 × 10^−4^	4.62 × 10^−5^	0	2.57 × 10^−4^
	90	Total mutations/bases sequenced	7/16276	4/40404	14/18252	26/55848	4/21216	4/21060	5/15288
		Mutation frequency	4.30 × 10^−4^	9.90 × 10^−5^	7.67 × 10^−4^	4.66 × 10^−4^	1.88 × 10^−4^	1.90 × 10^−4^	3.27 × 10^−4^
MED	0.5	Total mutations/bases sequenced	5/17528	7/43512	4/19656	9/60144	4/22848	6/22680	1/16464
		Mutation frequency	2.85 × 10^−4^	1.61 × 10^−4^	2.03 × 10^−4^	1.50 × 10^−4^	1.75 × 10^−4^	2.64 × 10^−4^	6.07 × 10^−5^
	15	Total mutations/bases sequenced	164/15024	84/37296	10/16848	154/51552	164/19584	74/19440	18/14112
		Mutation frequency	1.09 × 10^−2^	2.25 × 10^−3^	5.93 × 10^−4^	2.98 × 10^−3^	8.37 × 10^−3^	3.81 × 10^−3^	1.28 × 10^−3^
Asia II 1	0.5	Total mutations/bases sequenced	4/14711	5/36519	1/16497	6/50478	3/19176	1/19035	3/13818
		Mutation frequency	2.72 × 10^−4^	1.37 × 10^−4^	6.06 × 10^−5^	1.12 × 10^−4^	1.56 × 10^−4^	5.25 × 10^−5^	2.17 × 10^−4^
	15	Total mutations/bases sequenced	108/10955	76/27195	3/12285	76/37590	109/14280	37/14175	1/10290
		Mutation frequency	9.85 × 10^−3^	2.79 × 10^−3^	2.44 × 10^−4^	2.02 × 10^−3^	7.63 × 10^−3^	2.61 × 10^−3^	9.72 × 10^−5^

### Types of mutations in *B. tabaci* MED whiteflies

All the mutations identified in the TYLCV progeny populations were nucleotide substitutions rather than indel. Further analysis of these mutations showed that there were major biases in each TYLCV progeny population. In the TYLCV progeny populations obtained from TYLCV-infected tomato plants, as well as the populations from *B. tabaci* MED whiteflies collected at 12 h AAP, transitions of C to T, G to A, or transversion of G to T were dominant compared with the other types of mutational changes (Table 3). Transversions of A to C, C to G, and T to G were less often found in the TYLCV progeny populations from TYLCV-infected tomato plants and *B. tabaci* MED whiteflies collected at 12 h AAP. The C to T, T to C, A to G transitions, and a T to G transversion were, however, the main changes in the populations from *B. tabaci* MED whiteflies collected at 15 d after being transferred to cotton plants (Table 3). These findings indicate that there are major substitution biases in the populations from *B. tabaci* MED whiteflies fed on cotton plants compared with those obtained from TYLCV-infected tomato plants or from *B. tabaci* MED whiteflies collected at 12 h AAP.

**Table 3:** Nucleotide substitutions in TYLCV populations obtained from the TYLCV-infected tomato plant and viruliferous *B. tabaci* MED and *B. tabaci* Asia II 1.

Host	Days p.i.	Original base	Resulting base^(a/b)^			
			A	T	C	G
Tomato	30	A	—	2/2	0	1/1
		T	1/1	—	0	0
		C	5/5	9/9	—	0
		G	6/6	5/5	1/1	—
	45	A	—	0	0	3/3
		T	1/1	—	6/9	3/3
		C	1/2	5/5	—	0
		G	4/5	10/10	0	—
	90	A	—	1/1	1/1	4/5
		T	0	—	4/4	1/2
		C	4/5	11/13	—	1/1
		G	8/11	8/8	1/1	—
MED	0.5	A	—	1/1	0	0
		T	0	—	0	0
		C	3/5	13/15	—	0
		G	3/16	2/2	0	—
	15	A	—	3/17	3/4	7/104
		T	3/32	—	10/16	4/107
		C	4/19	17/76	—	4/7
		G	8/47	5/36	2/45	—
Asia II 1	0.5	A	—	0	0	1/1
		T	1/1	—	2/2	0
		C	2/2	5/5	—	1/1
		G	5/38	2/2	1/1	—
	15	A	—	1/2	1/1	2/68
		T	2/35	—	0	5/106
		C	1/1	9/45	—	1/1
		G	1/1	3/3	1/35	—

^a/b^The values means the number of each transition or transversion observed in each progeny population. ^a^Represents the mutations occurring in the same nucleotide position were grouped and counted as one site. ^b^Represents the mutations in the same nucleotide position were ungrouped.

### Genetic variation of TYLCV populations in *B. tabaci* Asia II 1 whiteflies

To investigate the role of different species of whiteflies on the genetic variability of TYLCV populations, full-length TYLCV sequences were amplified from indigenous *B. tabaci* Asia II 1 whiteflies collected either at 12 h AAP or at 15 d after being transferred from infected tomato plants to cotton plants. Similar to what was observed for *B. tabaci* MED whiteflies, the mutation frequency in TYLCV populations obtained from *B. tabaci* Asia II 1 whiteflies collected at 12 h AAP was about 1.64 × 10^−4^ (Table 1). The mutation frequency increased to 3.44 × 10^−3^ after the whiteflies were transferred to cotton plants (Table 1).

Analysis of mutation frequency in the TYLCV populations from *B. tabaci* Asia II 1 whiteflies showed that mutation frequencies in the V1, C1, C2 and C3 ORFs increased to 10^−3^ after the whiteflies were transferred to cotton plants (Table 2). Mutation distribution analysis showed that the increased mutation frequency was due mainly to nucleotide substitutions at multiple distinct positions (nucleotide positions 38, 138, 778, 828, 1110, 1359, 1559, 1944, and 2689) (Fig. 1), which accounted for 92.53% of the total mutations. Strikingly, most of these mutations occurred simultaneously in the same clone, and distinct positions were also observed in the populations from *B. tabaci* MED whiteflies collected at 15 d after being transferred to cotton plants,. Substitution pattern analysis showed a bias of C to T and A to G transitions and T to G transversion (Table 3). These results suggest that both species of *B. tabaci* influenced the genetic variability of TYLCV.

### Mutagenesis of the mutational hot spots reduced the pathogenicity of TYLCV

**Figure 2:**
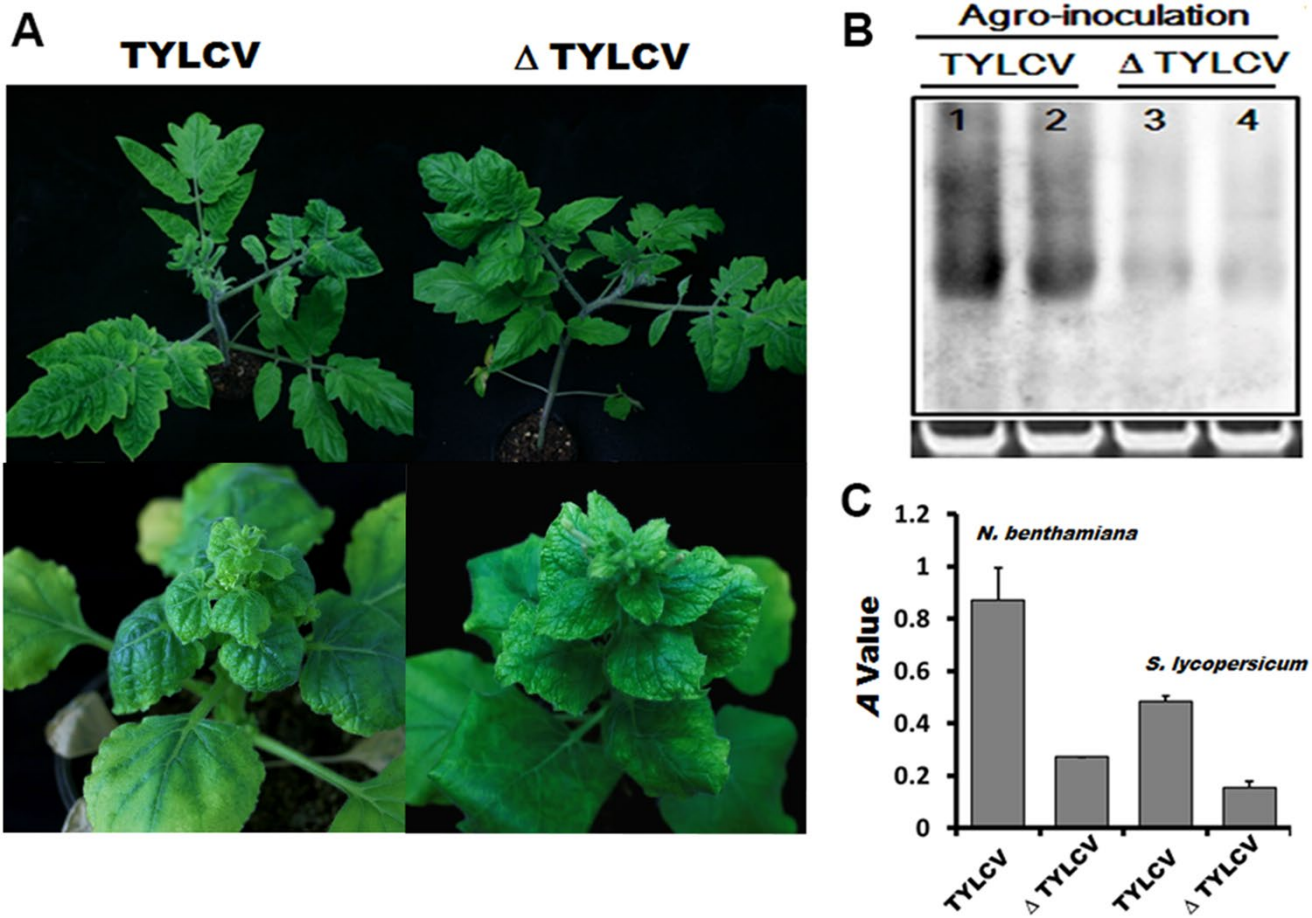
Agroinoculation-mediated test of the pathogenicity of TYLCV and TYLCV mutant. (**A**) TYLCV mutant attenuates the symptoms induced by TYLCV. Tomato and *N. benthamiana* plants were agro-inoculated with TYLCV or TYLCV mutant (Δ TYLCV), and were photographed 30 days later. (**B**) DNA gel blot hybridization analysis of TYLCV DNA from systemically infected tomato leaf tissues. 20 μg of total DNA was used for each lane. An ethidium bromide stained gel was provided as a loading control. (**C**) Triple antibody sandwich ELISA test of TYLCV viral content in TYLCV or TYLCV mutant-infected tomato and *N. benthamiana* plants, respectively. *A* value represents the mean value obtained from four independent plants with three replicates at OD_405_. The error bars indicate the standard deviation of each sample. *p < 0.05.

The mutational hot spots of TYLCV in both species of *B. tabaci* led us to investigate the biological significance of these mutations. One of the full-length clones containing simultaneous mutations at nucleotide positions of 38, 138, 778, 828, 1110, 1112, 1559, 1944, and 2689 was chosen for the construction of an infectious clone containing a 1.7-mer tandem repeat of the mutated TYLCV genome. Agroinoculation of the mutated infectious clone into tomato and *N. benthamiana* plants showed that the TYLCV mutant was infectious in these plants, producing yellowing, leaf curling, and dwarfing symptoms. The symptoms in both plants, however, were milder when compared to those induced by wild-type TYLCV (Fig. 2A). Reduced virus concentration was found in TYLCV mutant-infected tomato and *N. benthamiana* plants by triple antibody sandwich ELISA (Fig. 2C). Southern blot analysis of DNA isolated from infected tomato leaves showed that tomato plants agroinoculated with the TYLCV mutant accumulated less viral DNA compared to the wild-type virus (Fig. 2B). Sequence analysis confirmed that each mutation was maintained in systemically infected tomato leaves at 30 dpi and 45 dpi.

### TYLCV mutant could be transmitted by whitefly

**Figure 3:**
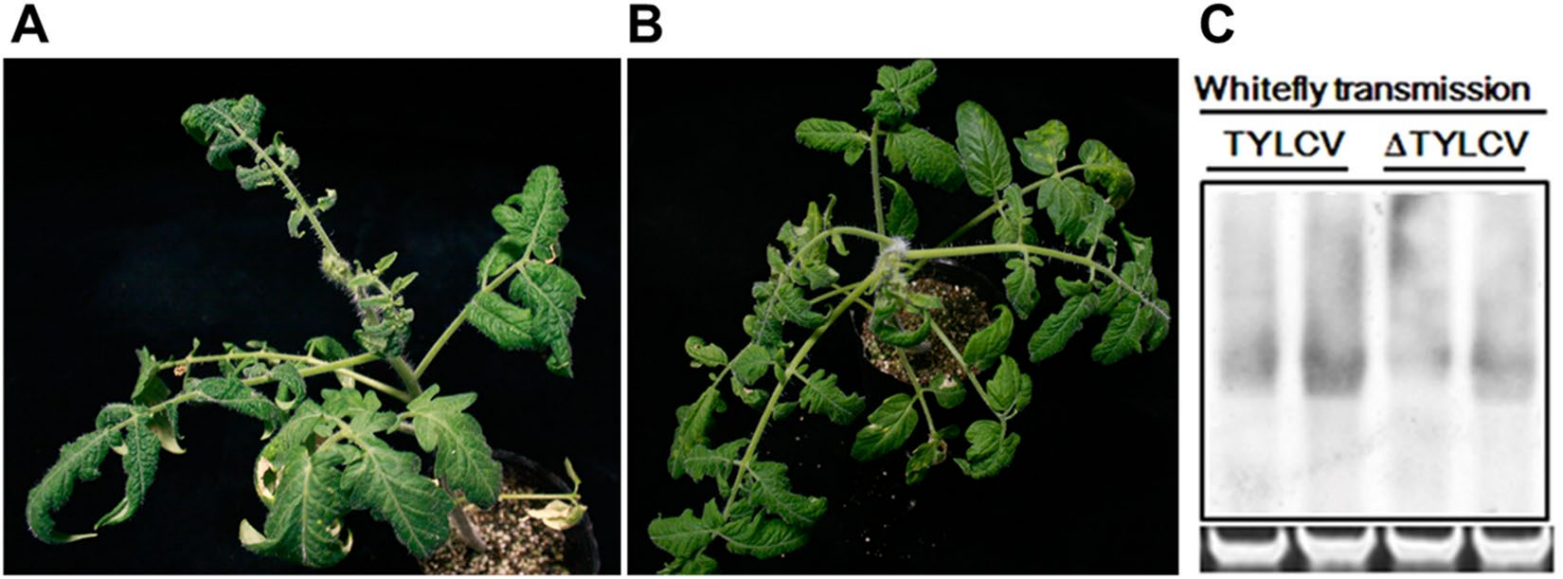
TYLCV (**A**) and TYLCV mutant (**B**) could be transmitted by the whitefly *B. tabaci* MED. (**C**) Detection of TYLCV DNA from tomato leaf tissues transmitted by whitefly at 30 days.

To further understand the ability of the TYLCV mutant to be transmitted by *B. tabaci*, tomato plants inoculated with wild-type TYLCV or the TYLCV mutant at 30 dpi were used as individual source tissues for *B. tabaci* MED feeding. To ensure the acquisition of the TYLCV mutant by whiteflies, ten *B. tabaci* MED whiteflies fed on TYLCV mutant-infected tomato plants were randomly selected at 12 h AAP and the presence of viral DNA was verified by PCR. The percentage of detectable viral DNA reached 100% after a 12 h AAP for the *B. tabaci* MED (data not shown). After the AAP, transfer of *B. tabaci* MED to healthy tomato plants caused symptoms similar to those induced by agroinoculation of the mutant viral infectious clone (Fig. 3B). Southern blot analysis showed that the TYLCV mutant could be detected in systemically infected leaves, indicating that the mutant can be transmitted by the whiteflies (Fig. 3C).

## Discussion

*B. tabaci* is currently considered to be a cryptic species complex containing at least 35 morphologically indistinguishable species^22,23^. *B. tabaci* whiteflies are important and efficient transmission vectors for viruses in the genus *Begomovirus*. They also cause severe damage to crops through direct feeding on plants, induction of various phytotoxic disorders and excretion of large amounts of honeydew on its host plants^24,25^. Begomoviruses are known to be transmitted by whiteflies. The interplay between whiteflies and begomoviruses, including the factors required for virus acquisition and transmission by the vector, and the effects of begomoviruses on the fecundity and longevity of whiteflies have been intensively studied in recent years^26^. Findings from these studies provided useful information on the understanding of begomovirus-whitefly interactions. In this study, we compared the nucleotide sequences of TYLCV populations obtained from either whiteflies or from the host tomato plant. Our results indicate that TYLCV populations from whiteflies differ significantly from those obtained from TYLCV-infected tomato plants, providing new information on the dynamics and evolution of TYLCV populations in its transmission vector.

Analysis of tomato yellow leaf curl China virus (TYLCCNV) populations obtained from a naturally infected tomato plant as well as TYLCCNV-inoculated *N. benthamiana* and tomato plants showed that the structure of the TYLCCNV population was quasispecies-like, and that rapid accumulation of variation during TYLCCNV infection generated diversity levels comparable to those reported for plant RNA viruses^13^. In this study, we determined that the mutation frequency of TYLCV was approximately 10^−4^ in TYLCV-infected tomato samples harvested at 30, 45 and 90 dpi. Considering the very low mutation frequency (1.8 × 10^−5^) found during the initial rolling circle amplification assay using the phi29 DNA polymerase, it is reasonable to believe that the mutation frequency for TYLCV populations from infected tomato is similar to that reported for TYLCCNV and many other RNA viruses. This finding suggests the existence of a quasispecies-like phenomenon in the TYLCV populations from infected tomato plants.

Our data also showed that the TYLCV mutation frequency was approximately 10^−3^ for both invasive *B. tabaci* MED and indigenous *B. tabaci* Asia II 1 whiteflies collected at 15 d after being transferred to cotton plants. Statistical analysis indicates that these mutation frequencies are significantly higher than those observed in TYLCV populations obtained from infected tomato plants. The presence of diverse begomovirus populations in infected field and greenhouse host plants was reported previously by multiple research groups^13–15,27^. The data presented here show, for the first time, the presence of highly diversified populations of TYLCV in its transmission vector.

Composition analysis of TYLCV progeny populations from *B. tabaci* MED and *B. tabaci* Asia II 1 whiteflies showed that the high mutation frequency of TYLCV in its transmission vectors was due mainly to mutations occurring coincidentally at several distinct sites. Introduction of these mutations in the infectious clone of TYLCV showed that they did not affect its ability to be transmitted by whitefly, but decreased its pathogenicity in both tomato and *N. benthamiana*. Further analysis of the nine mutations showed that three of the nine mutations impact the protein translation. The C-to-T change at 828 in the V1 ORF results in the change of proline to leucine. The T-to-A and G-to-T change at 1110 and 1112 in the C2 ORF leads to the change of threonine to serine, and alanine to glutamate, respectively. In future studies, it will be interesting to reintroduce these three mutations into the TYLCV genome to elucidate their functional consequences.

The molecular mechanism of these frequently occurred mutations in the whitefly *B. tabaci* is yet to be determined. One possible explanation is that highly sensitive Phi29 DNA polymerase amplified the poor genomic DNA template that resulted from autophagy-mediated resistance to TYLCV infection in whiteflies^28^. However, the number of mutations occurring at each of these distinct sites varied, ruling out the possibility that the mutant sequences obtained would come from the same DNA template. The other potential explanation for the present results is that bottlenecks imposed by the transmission vector might be the major force driving the variability of TYLCV. Previous studies have established that bottlenecks associated with vector-mediated transmission or colonization to a new host or organ, are common for populations of plant RNA viruses. These bottlenecks are implicated in influencing the maintenance of the changes in viral genomes and the fitness of viral populations. Viral populations with a high degree of variability are continuously generated over the life cycle of viruses. After a bottleneck, individuals of viral progeny can be stochastically reduced regardless of their fitness or any phenotypic traits. If bottleneck size is narrow, fewer viral variants pass through the bottleneck and the variation frequency increases. As the bottleneck size increases, there are few single-variant infections, and the frequency of virus variant in infected hosts decreases^29^. When the effective population size is large, viral populations will gain fitness due to the quickly increased frequency of competitive genomes containing reversions or second-site compensatory mutations. However, in small populations, the presence of bottlenecks may result in fitness decline due to the inability to create compensatory mutations^29^. Fitness declines resulting from repeated genetic bottlenecks have been investigated in several plant virus systems, such as cucumber mosaic virus and tobacco etch virus^6–8^. In this study, simultaneous introduction of the nine mutations decreased the pathogenicity of TYLCV in both tomato and *N. benthamiana*. It is probable that the variant might be caused by mutation and raised to a high frequency by drift due to the narrow bottleneck in insect, thus the overall fitness of a viral population is fluctuated depending on a stochastic process.

Another interesting question is how the mutations can be generated in the whitefly. One potential possibility is that they are generated during viral replication. TYLCV is transmitted by the whitefly *B*. *tabaci* in a persistent, circulative manner. Although kinetic quantification of the accumulation of both complementary and virion sense viral DNA molecules within *B. tabaci* using two step qPCR procedure showed no significant increase of viral DNA accumulation in the insect vector^30^, an increased amount of viral DNA corresponding to genes from both strands of TYLCV was detected by real-time RT-PCR in individuals of *B. tabaci* following 8 h of AAP on TYLCV-infected tomato plants or purified virions and then transferred to non TYLCV-host cotton plants^31^. Accumulation of viral DNA and viral transcripts was also observed using Fluorescence *in situ* hybridization analysis^31^. Interestingly, localization of the complementary viral genome (present on the viral genome replicates) was targeting to the nuclei of midgut epithelial cells. These sites differed from those illuminated by the viral strand probe, which were limited mostly to the filter champer and ceca and less frequently to the descending and ascending midguts, implying for the formation of a viral dsDNA replicative form and the sites of virus replication^31^. The immunodetection of CP further indicated for the formation of newly-assembled virions^31^. Moreover, levels of viral DNA can also increase continuously or decrease after pesticide or heat stresses were imposed to whiteflies, respectively^31^. These results provided different evidences supporting the replication of TYLCV in whitefly. We could also not rule out the alternative possibility that cellular chaperones or chaperonins encoded by secondary endosymbionts of *B. tabaci* contribute to the mutations. It has been shown that most viruses need heat shock proteins to solve their protein-folding problems or to interfere with cellular processes^32^. Expression of Hsp70 can be induced by the presence of mutant misfolded coat protein of tobacco mosaic virus rather than the wild-type CP^33^. The role of GroEL chaperonins in the transmission efficiency of TYLCV has also been well illustrated^34^, however, their potential role in virus mutation needs to be further investigated.

Genetic variability is thought to be critical for the survival of viruses, facilitating adaptation to ever-changing environments and hosts. Since begomoviruses are transmitted by whiteflies in nature, it is still unknown whether the genetic variation of TYLCV in whiteflies could be restored or some fit mutations remained after being transmitted to new host plants. Further experiments using next-generation sequencing will need to be performed to determine the effect of vector transmission on viral genetic variability.

In conclusion, our data constitute the first report of genetic variability of a begomovirus in its insect vector. There must be a complex interplay between the various evolutionary processes to confer the emergence of viral populations in nature. Further research to improve our understanding of begomovirus evolution will increase insights into the dynamics and mechanisms of the long-term evolution of viruses in nature.

## Methods

### Sources of whiteflies, host plants, and virus, and virus inoculation

Both invasive *B. tabaci* MED and indigenous *B. tabaci* Asia II 1 whiteflies were maintained on *G. hirsutum* (cotton) cv. Zhe-Mian plants grown inside an insect-proof chamber set at 27 ± 1 °C, 70 ± 10% relative humidity and 14/10 h (day/night) photoperiod. The two different *B. tabaci* species were determined using random-amplified polymorphic DNA polymerase chain reaction (RAPD-PCR) together with sequencing of the mitochondrial cytochrome oxidase 1 gene as described previously^35,36^.

A previously constructed infectious TYLCV clone (TYLCV-[SH2])^18^ was used as the source of inoculum. *S. lycopersicum* (tomato) cv. Hongbaoshi plants at the 4 to 5 true-leaf stage were agroinoculated directly with the infectious TYLCV clone as described previously^37^. The inoculated plants were grown inside the insect-proof chamber and observed for TYLCV symptom development for 90 days.

### Acquisition of TYLCV by *B. tabaci* MED and *B. tabaci* Asia II 1 whiteflies

After 30 days post inoculation, tomato plants showing typical TYLCV symptoms were used as virus source for the acquisition assay. Three independently caged TYLCV-infected tomato plants were used for each of the two *B. tabaci* species, *B. tabaci* MED and *B. tabaci* Asia II 1. In each acquisition assay, approximately 300 whiteflies representing one of the two species were allowed to feed on a single caged TYLCV-infected tomato plant for 12 h. After AAP, approximately half of the whiteflies were randomly collected and frozen in liquid nitrogen. The other half of the whiteflies were transferred gently onto healthy cotton plants. Tomato plants were then fumigated with imidachloprid, and placed in a separate cage. After 15 days feeding on the cotton plants, whiteflies were collected immediately and frozen in liquid nitrogen, and then stored at −80 °C until use.

### Nucleic acid extraction

Nucleic acids were extracted from individual whitefly samples as described previously by Luo *et al*.^35^. TYLCV-infected tomato plants were sampled at 30, 45 and 90 days post inoculation (dpi), respectively. DNAs from the harvested tomato leaf tissues (0.1 g tissue/sample) were extracted as described previously by Zhou *et al*.^38^.

### Isolation, cloning and sequencing of full-length TYLCV sequences

Viral DNAs from each randomly picked whitefly or infected tomato plant were cloned separately and treated as unique populations. Circular full length TYLCV genome was rolling-circle amplified either from 1 μg of tomato plant DNA or 2 μL of nucleic acid extractions from whiteflies using the high-fidelity phi29 DNA polymerase (TempliPhi^TM^, GE Healthcare, Bucks, UK) as previously described^39^. The amplified concatemers were digested with *Sac*I restriction enzyme to produce linearized full-length viral genome of approximately 2.8 kb. The resulting TYLCV DNAs were individually ligated into the pGEM-7ZF+ vector (Promega, Madison, WI) previously digested with *Sac*I. Approximately twenty colonies were picked up randomly from each plate and plasmid DNAs were prepared individually. Full length TYLCV sequences of these clones were determined using an automated DNA sequencer (Model 3730, Applied Biosystems) using the M13 forward and reverse primers, and a pair of walking primers TYLCV-W/F (5′-TCTGCAATCCAGGACCTACC-3′, positioned from bases 1717 to 1698) and TYLCV-W/R (5′-AGTCTATCTTGCAATATGTG-3′, positioned from bases 133 to 152) as previously described^20^. The resulting sequences were edited and assembled using Lasergene version 7 (DNASTAR, Inc.). The fidelity of rolling circle amplification was determined by sequencing 20 clones generated from the RCA products using the TYLCV infectious clone as a template. Only one of these clones contained a single mutation. Thus, the experimental procedure of rolling-circle amplification was estimated to be 1.8 × 10^−5^.

### Sequence analysis

The circular genomic sequences were arranged so that they all started from the nick site in the invariant nonanucleotide sequence at the origin of replication (TAATATT-3′/5′-AC)^40^. Multiple sequence alignments were then generated using MUSCLE (http://www.drive5.com/muscle/)^41^. The parental TYLCV sequence (AM282874) was considered as the ancestral sequence. Individual clones from an individual sample were compared with the ancestral sequence and each clone containing one or more nucleotide changes was considered as a mutant clone, and each single nucleotide change was regarded as a mutation. Variability of viral populations was shown as the percentage of mutant clones and the mutation frequencies, which were estimated as the total number of mutations observed in all clones from an individual population divided by the total number of bases sequenced for that population.

### Construction and infectivity of infectious clone of mutant TYLCV

To generate a 1.7-mer tandem repeat of the TYLCV mutant, one of the full-length clones containing mutations at nucleotide positions of 38, 138, 778, 828, 1110, 1112, 1559, 1944, and 2689 (designated as pGEM-ΔTYLCV) was used as a template. PGEM-ΔTYLCV was digested with SacI and XbaI and the 2.1 kb fragment was introduced into the binary vector pBinPLUS to produce pBinPLUS-ΔTYLCV 0.7 A. Then, the full-length SacI-digested fragment of pGEM-ΔTYLCV was inserted into the unique SacI site of pBinPLUS-ΔTYLCV 0.7 A to produce clone pBinPLUS-ΔTYLCV 1.7 A. The resultant clone was subsequently electroporated into *Agrobacterium tumefaciens* strain EHA105 and used to evaluate its infectivity in tomato and *N. benthamiana* plants as described^37^.

### Whitefly transmission

Whitefly transmission experiments were performed using the *B. tabaci* MED whiteflies. Tomato plants infected with wild-type or mutant TYLCV were used as sources of inoculum for whitefly transmission as previously described^42^. For each transmission assay, 200 whiteflies were fed on a single caged tomato plant for an AAP of 12 h. After that, whiteflies were gently transferred to five tomato plants for an inoculation access period of 48 h. Tomato plants were then fumigated with imidachloprid, and placed in a closed cage. Viral infection was evaluated by symptom observation and Southern hybridization analysis at 30 days post transmission.

### Analysis of viral DNA in plants

Genomic DNA was isolated from newly emerging leaves of tomato plants as described^38^, and fractionated by 1% (w/v) agarose gel electrophoresis in TBE buffer. Southern hybridization to assess viral DNA accumulation was carried out essentially as described^18^, except that hybridization signals were detected using the anti-digoxigenin AP chemiluminescent substrate CSPD following the manufacturer’s instructions (Roche Diagnostics, Mannheim, Germany). Hybridization signals were detected using ImageQuant 4000 (GE healthcare). All leaves were collected at 30 days after agro-inoculation or whitefly transmission.

### Serological tests

Triple antibody sandwich enzyme-linked immunosorbent assay (ELISA) was carried out using monoclonal antibody raised against TYLCV as described^43^.

## Electronic supplementary material

Dataset 1
